# Non-Invasive In Vivo Study of the *Trypanosoma vivax* Infectious Process Consolidates the Brain Commitment in Late Infections

**DOI:** 10.1371/journal.pntd.0001976

**Published:** 2013-01-03

**Authors:** Simon D'Archivio, Alain Cosson, Mathieu Medina, Thierry Lang, Paola Minoprio, Sophie Goyard

**Affiliations:** 1 Institut Pasteur, Laboratoire des Processus Infectieux à Trypanosoma, Department of Infection and Epidemiology, Paris, France; 2 Institut Pasteur, Laboratoire d'Immunophysiologie et Parasitisme, Department of Parasitology, Paris, France; Fundaçao Oswaldo Cruz, Brazil

## Abstract

*Trypanosoma vivax*, one of the leading parasites responsible for Animal African Trypanosomosis *(Nagana)*, is generally cyclically transmitted by *Glossina spp.* but in areas devoid of the tsetse flies in Africa or in Latin American countries is mechanically transmitted across vertebrate hosts by other haematophagous insects, including tabanids. We followed on from our recent studies on the maintenance of this parasite *in vivo* and *in vitro*, and its genetic manipulation, by constructing a West African IL1392 *T. vivax* strain that stably expresses firefly luciferase and is fully virulent for immunocompetent mice. We report here on a study where murine infection with this strain was monitored *in vivo* using a non-invasive method. Study findings fully support the use of this strain in the assessment of parasite dynamics *in vivo* since a strong correlation was found between whole body light emission measured over the course of the infection and parasitemia determined microscopically. In addition, parasitemia and survival rates were very similar for mice infected by the intraperitoneal and sub-cutaneous routes, except for a longer prepatent period following sub-cutaneous inoculation with the parasite. Our results clearly show that when administered by the subcutaneous route, the parasite is retained few days in the skin close to the inoculation site where it multiplies before passing into the bloodstream. *Ex vivo* bioluminescence analyses of organs isolated from infected mice corroborated our previous histopathological observations with parasite infiltration into spleen, liver and lungs. Finally, our study reinforces previous observations on the presence of the parasite in the central nervous system and consequently the brain commitment in the very late phases of the experimental infection.

## Author Summary

Very little work has been performed on *Trypanosoma vivax* for decades, but the recent development of murine infection models and axenic cultures has enabled the genetic manipulation of this parasite and has opened the door to a more in-depth understanding of its biology and the infectious process that leads to animal trypanosomosis. We report herein the characterization of a luciferase-expressing strain that can be used to follow parasite dynamics *in vivo* in real time using a non-invasive method. Regardless of the inoculation parasite route and some minor differences concerning the length of the prepatent period of infection following the subcutaneous injection of the parasites, we highlight the general commitment of the organs triggered by the infection and particularly the presence of the parasite in the brain at late phases of disease. The study presented herein provides new insights into the interaction between *T. vivax* and its mammalian host and assesses new tools for *in vivo* drug screening.

## Introduction

Animal African trypanosomosis (AAT) is a major protozoan disease due to trypanosomes. The disease which is endemic in Africa is mainly caused by *Trypanosoma vivax, T. congolense* and *T. b. brucei*. *T. vivax* accounts for up to half of all AAT prevalence in West Africa where it is considered to be the major pathogen that together with *T. congolense* causes 3 million cattle deaths annually [1]–[3]. Furthermore, *T. vivax* but also *T. equiperdum and T. evansi* trigger different pathologies (*Nagana, Dourine* and *Surra*, respectively) and are species that have spread to South America. Globalization and livestock trade between countries, coupled with the lack of rapid diagnostic tools and the transport of infected animals to non-endemic areas have a huge impact on agriculture and, consequently, on the economy of breeding and endurance.

One of the specificities of *T. vivax* compared to other animal trypanosomes (*i.e. T. brucei spp* and *T. congolense*) is its ability to be transmitted not only by *Glossina spp.* (tsetse) flies but also by other biting flies of the *Tabanidae* and *Muscidae* families that can mechanically transmit the parasite among mammalian hosts [4], [5]. It is noteworthy that *Glossina spp.* are the only vectors in which *T. vivax* is able to multiply and pursue its differentiation into metacyclic forms. In contrast, *T. vivax* is unable to grow or multiply in other insects that can only mechanically transmit the parasite. Regardless of the natural type of transmission (cyclical or mechanical), *T. vivax* is inoculated in the subcutaneous tissue and the infective forms join the bloodstream via the lymphatic system. After one or more parasitemia peaks, the animals generally show neurological disorders in late phases of infection and perish [6], [7]. Ruminants and equines infected with *T. vivax* show a range of tissue damage and the diversity of the pathognomonic signs and the severity of the disease frequently correlate with the degree to which the host shows resistance (“tolerance”) or susceptibility to the parasite. Few studies have been conducted to compare the infective process following a bite by tsetse or tabanids, or experimental infections by intraperitoneal or subcutaneous inoculation routes [8].

In efforts to overcome the problems encountered when studying *T. vivax* infection and pathology in the field, we recently developed murine models that deliver sustained and reproducible infections which successfully mimic the parasitological, histological and pathological features of the infection and closely resemble those observed in cattle trypanosomosis [9], [10]. For instance, histopathological examinations performed throughout the infective process showed many necrotic foci in lymphoid and non-lymphoid organs with extravasated blood cells and trypanosomes in hemorrhagic spots. Most importantly, the infection resulted in multifocal lesions in the central nervous system along with vasogenic edema and damaged blood vessels characteristic of the late-stage ischemic necrosis caused by the wild-type strain. Although the presence of trypanosomes in the meningeal blood vessels at these late stages was suggestive of blood-brain barrier crossing and invasion of cells and parasites into the brain parenchyma [9], our knowledge of the invasive characteristics of *T. vivax*, its tissue tropism, the temporal course of its invasion and the crucial question of the permeabilization of the blood brain barrier is still incomplete.

In order to address some of these questions and supplement the conventional anatomic pathology examinations conducted during studies of the infectious process, we took full advantage of the latest advances made in *T. vivax* genetic manipulation [11] and engineered a parasite strain that stably expresses firefly luciferase. Here we report on the *in vitro* and *in vivo* characterization of the *T. vivax* luciferase strain and the validation of real-time biophotonic detection systems employed to study the propagation of this parasite *in vivo*. We determined method limits of detection and linearity ranges to better correlate mouse parasitemia with luminescence measured *in vivo*, and analyzed the course of the infection and parasite tissue distribution over time. Finally, we compared infection dynamics and organ commitment after subcutaneous and intraperitoneal inoculations with the parasite. Our results confirmed the usefulness of real-time biophotonic analysis in the study and monitoring of the *T. vivax* infectious process *in vivo*. Irrespective of the causes that have conducted each mouse to perish during early phases of infection, such as anemia, hyperparasitemia or organ failure, our data provide important evidence that at long-term trypanosomes attain the central nervous system of all the animals which have showed a extended survival just some days before death.

## Materials and Methods

### Ethics statement

All mice were housed in our animal care facility in compliance with European animal welfare regulations. Institut Pasteur is a member of Committee #1 of the *Comité Régional d'Ethique pour l'Expérimentation Animale* (CREEA), Ile de France. Animal housing conditions and the procedures used in the work described herein were approved by the “*Direction des Transports et de la Protection du Public, Sous-Direction de la Protection Sanitaire et de l'Environnement, Police Sanitaire des Animaux”* under number B 75-15-28, in accordance with the Ethics Charter of animal experimentation that includes appropriate procedures to minimize pain and animal suffering. PM is authorized to perform experiments on vertebrate animals (license #75-846 issued by the Paris Department of Veterinary Services, DDSV) and is responsible for all the experiments conducted personally or under her supervision as governed by the laws and regulations relating to the protection of animals.

### *T. vivax* parasite strain

*Trypanosoma (Dutonella) vivax* IL 1392 was originally derived from the Zaria Y486 Nigerian isolate [12]. These parasites have recently been characterized and are maintained in the laboratory by continuous passages in mice, as previously described in detail [9]. Seven to ten week-old male Swiss Outbred mice (CD-1, RJOrl:SWISS) (Janvier, France) were used in all experiments. They were injected intraperitoneally or sub-cutaneously with bloodstream forms of *T. vivax* (10^2^ parasites/mouse). Parasitemia was determined as previously described [9]. All animal work was conducted in accordance with relevant national and international guidelines (see above).

### *In vitro* luciferase assay

A luciferase assay kit (Roche Molecular Biochemicals; Mannhein, Germany) was used to monitor luciferase expression. Serial dilutions of parasite suspensions were washed in PBS and pellets were suspended in 150 µl of cell lysis buffer. The lysates were then transferred into white, 96-well microplates (Dynex Technologies, Chantilly, France). Light emission was initiated by adding the luciferin-containing reagent, in accordance with manufacturer instructions. The plates were immediately transferred to the luminometer (Berthold XS^3^ LB960; Thoiry, France) and light emission was measured for 0.1 s. Luminescence was expressed in Relative Light Units (RLU).

### *In vivo* bioluminescence imaging

Mice were inoculated intraperitoneally with luciferin (D-Luciferin potassium salt, Xenogen, California), the luciferase substrate, at a dose of 150 mg/kg before any bioluminescence measurements were made. They were anaesthetized in a 2.5% isoflurane atmosphere (Aerane, Baxter SA, Maurepas, France) for 5 minutes and kept in the imaging chamber for analysis. Emitted photons were acquired for 1 minute by a charge couple device (CCD) camera (IVIS Imaging System Lumina, Caliper, Villepinte, France) set in high resolution (medium binning) mode. The analysis was then performed after defining a region of interest (ROI). The same ROI was used for all animals and all time points. Total photons emitted from the image of each mouse were quantified using Living Image software (Xenogen Corporation, Almeda, California), and results were expressed as number of photons/sec/ROI.

## Results

### Epimastigote forms of *T. vivax* show more bioluminescence than bloodstream forms

**Figure 1:**
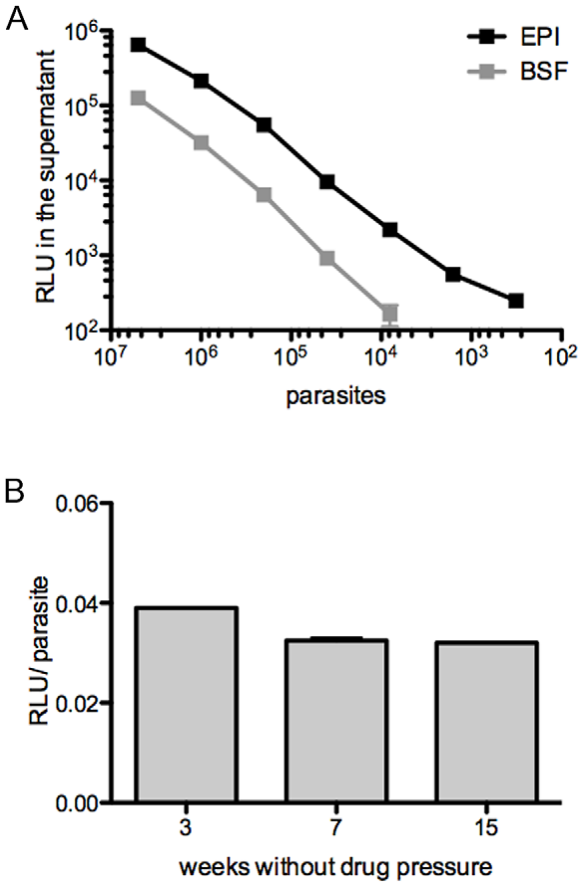
Characterization of bioluminescence production by epimastigotes and bloodstream evolutive forms of *Tv*LrDNA-luc strain. **A**. Serial dilutions of *Tv*LrDNA-luc epimastigotes (EPI, black squares) or bloodstream forms purified from blood (BSF, gray squares) were measured *in vitro* for bioluminescent activity expressed in RLU (Relative Light Unit); the results are representative of more than three independent experiments. **B**. Bioluminescence emission per *Tv*LrDNA-luc BSF without drug pressure selection; the results are expressed in RLU/parasite and correspond to arithmetic means +/− SD of the means; p>0,05.

The methods used to engineer this *T. vivax* strain that stably expresses firefly luciferase (*Tv*LrDNA-luc) have been described elsewhere [11]. The infective forms of these recombinant parasites maintain their infectivity in immunocompetent mouse strains and show the same parasitemia profiles over time and result in similar levels of mortality as wild type (WT) *T. vivax*. Luciferase expression levels in non infective epimastigote axenic forms (EPI) and in infective bloodstream trypomastigote (BSF) forms of *Tv*LrDNA-luc were compared by measuring the luciferase activity of equivalent numbers of parasites purified from axenic cultures or mouse blood, respectively. Serial dilutions of EPI and BSF were washed, lysed and the extract supernatants assayed in parallel for *in vitro* luciferase activity by measuring relative light emission (RLU) initiated by adding luciferin substrate. Figure 1A illustrates the linearity of the RLU results over more than 3 logs for both EPI and BSF *Tv*LrDNA-luc extract supernatants. Since no bioluminescence was detected in the WT EPI and BSF parasites included in the assays (<50 RLU), these results indicate that this light emission is specific to bioluminescent (luciferase-expressing) parasites.

Limits of detection were about 300 EPI and 8000 BSF using the bioluminescence assay. EPI clearly gave 7 to 10 fold the luciferase activity of purified BSF. To check that BSF serial passages *in vivo* do not result in any loss of the construction carrying the luciferase and the resistance marker genes, *Tv*LrDNA-luc parasite strain was maintained *in vivo* without drug pressure for 3, 7 or 15 sequential passages and compared for light emission. Figure 1B shows that light emission was comparable whatever the number of passages *in vivo*. The results obtained confirmed that the differences observed between EPI and BSF were not due to any *in vivo* loss of the construction. These differences therefore may reflect dissimilar stabilities of the enzyme at different temperatures or, like for other trypanosomatids [13], may stem from the distinct morphometries of EPI and BSF which are compatible with their size, nucleic acids and protein contents. Altogether, plasmid integration in the ribosomal region of *T.vivax* was shown to be stable over at least 15 consecutive passages, corresponding to 15 weeks.

### Validation of the *T. vivax* bioluminescent strain and determination of imaging parameters for analysis of the infectious process *in vivo*

**Figure 2:**
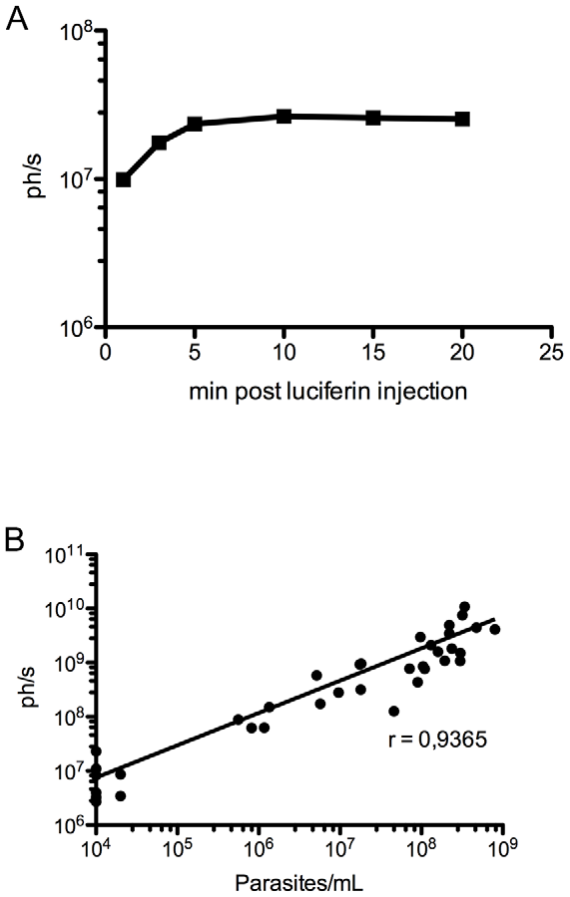
Correlation between parasitemia and total bioluminescence recorded from the entire infected animal. Male Outbred mice were injected intraperitonneally with 10^2^ *Tv*LrDNA-luc BSF. **A**. Total light emission kinetics following D-Luciferin IP injection in a mouse with 1.10^6^ parasites/mL. The figure depicts the data from 1 out of more than 34 independent mice analyzed individually **B**. Bioluminescence over a Region of Interest (ROI) including the entire animal body surface measured 10 minutes after D-Luciferin i.p. injection. The results are expressed individually for each measurement resulting from two groups of 17 mice. Parasitemia was determined by optical microscopy concomitantly with each bioluminescence measurement. Correlation factor was calculated using Spearman's non parametric correlation test r = 0.9365, p<0.0001.

Parasite dissemination and disease progression in a *T. vivax-*infected mouse model previously studied in the laboratory, was followed by using sensitive, non-invasive optical imaging to track *Tv*LrDNA-luc parasite strain in live mice. The bioluminescent signal obtained *in vivo* with *Tv*LrDNA-luc parasites was validated by considering criteria such as background spontaneous signals obtained from whole-body images of mice infected with WT parasites and optimal time period between substrate administration to live animals and image capture. Firstly, a group of mice were infected with WT parasites, injected with D-Luciferin and subsequently exposed to photon detection under the IVIS Lumina Imaging System (IVIS) to determine background emissions across the entire body. This resulted in 10^6^ ph./s being considered as the background level for further *in vivo* experiments using the *Tv*LrDNA-luc parasite strain (not shown). Secondly, another group of mice were infected with *Tv*LrDNA-luc parasite strain and analyzed once the infection had resulted in moderate parasitemia (10^6^ parasites/mL). Total body light emission was determined under the IVIS at time points 1, 3, 5, 10, 15 and 20 minutes after D-Luciferin injection. As can be seen in Figure 2A, light detection was maximal between 5 and 10 minutes after substrate injection and did not show any major variations up to 20 minutes after the injection. Further *in vivo* measurements were then made 10 minutes after the D-Luciferin injection. Lastly, we checked whether or not the light emitted correlated with the *T. vivax* infectious process *in vivo*. To do this, we determined whether or not the parasitemia observed microscopically correlated with the bioluminescence measured over the whole animal. Parasites were counted under a light microscope in five microliters of blood harvested individually from the tail vein of mice infected with 10^2^ *Tv*LrDNA-luc parasites, and parasitemia was expressed as number of parasites per mL of blood. Mice were immediately injected with D-Luciferin and submitted to whole-body imaging. An extensive and increasing light emission was observed during the course of the infection, as shown in Figure 2B. Total bioluminescence increased in the course of the infection and in line with the parasite count obtained optically, reaching ph./s levels that were more than 1000 fold the background level with parasitemia of 10^8^ parasites/mL. The exhaustive plotting of bioluminescence *versus* parasitemia depicted in Figure 2B represents 35 individual measurements obtained in a group of 15 mice and shows a close correlation between the 2 parameters, as confirmed by a high Spearman rank correlation coefficient of 0.9365. These findings validated the *Tv*LrDNA-luc bioluminescent strain and the baseline imaging parameters necessary to analyze and monitor *T. vivax* infection and disease progression *in vivo*.

### Impact of infection route on parasite burden and mouse survival

**Figure 3:**
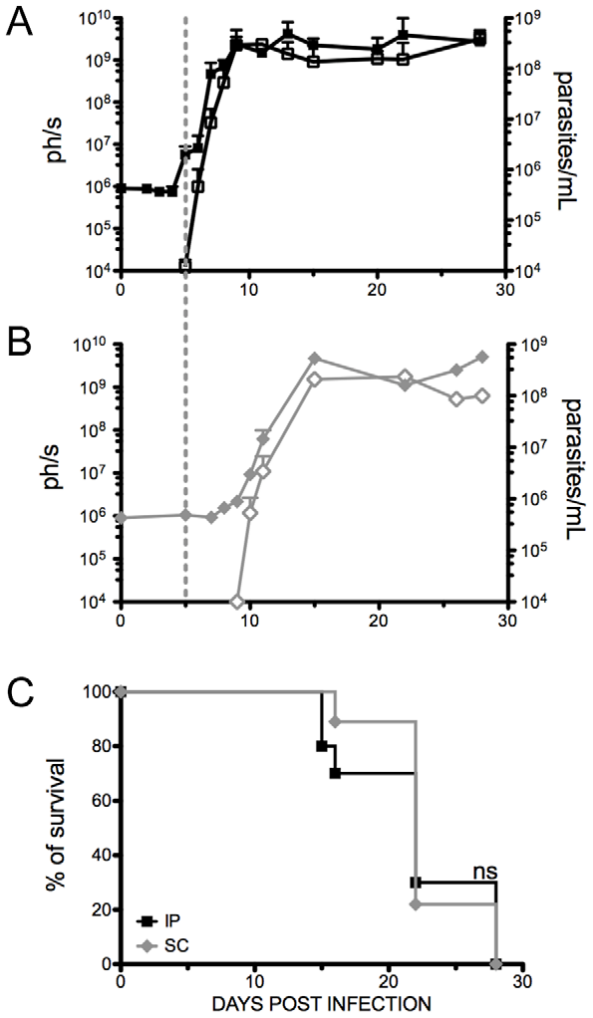
Mice injected by the subcutaneous route show a longer prepatent period than those injected intraperitoneally. Parasitemia (empty symbols) and total bioluminescence (full symbols) were determined in groups of mice injected with 10^2^ *Tv*LrDNA-luc parasites by (**A**) the intraperitoneal route (squares) or (**B**) the subcutaneous route (diamonds). The graphs depict the arithmetic means of values obtained individually for at least 3–6 mice per time point ± SD of the means. The results are representative of two independent experiments that used respectively 16 and 18 mice for the intraperitoneal route and 12 and 14 mice for the subcutaneous route. For a better comparison, the dashed vertical line that crosses the figures A and B illustrates the onset of microscopically-detectable parasitemia (10^4^/ml blood) for the IP injected group. **C**. Survival rates for the different infection routes. The cumulative mortality was recorded over time and survival curves are the result from the combination of the two independent experiments and Kaplan-Meir survival estimate curves were plotted. Only 3–6 mice (20–30%) are alive by the end of each of the two experiments but all die at day 28 of infection. Comparison between the curves was performed using the non parametric Mantel-Cox log-rank test. No significant difference was observed between the plots; p>0.5.

In order to gain a clearer insight into disease progression, and in particular determine whether *T. vivax* multiplication is confined to the vascular compartment, we compared the parasite dissemination after infection by two different routes. The conventional intraperitoneal (IP) route commonly used for mouse experimental infections was compared to the subcutaneous (SC) inoculation route that closely resembles the natural infections, cyclic or mechanically conveyed by the insects. The course of the resulting infection together with parasitemia and survival rates were therefore studied in mice infected subcutaneously or intraperitoneally with 10^2^ *Tv*LrDNA-luc BSF parasites. As can be seen in Figures 3A and 3B, no substantial differences were observed for parasitemia between the two groups. As expected, a straightforward correlation was found between the numbers of parasites determined optically and the signals resulting from bioluminescent parasites. The only difference found between the groups was the length of the prepatent period preceding the microscopically-detectable parasitemia (10^4^ parasites per ml of blood). As shown in Figure 3B, mice infected by the subcutaneous route showed a longer prepatent period (9 to 10 days) and consequently a more delayed onset parasitemia than mice inoculated by the intraperitoneal route that presented detectable parasites 5 days after infection (Figure 3A). Similar data were obtained after experimental infections with *T. congolense*, as reported previously [8]. With the exception of this time lag, parasite multiplication was seen to follow the same kinetics with both routes of infection and parasitemias were invariably similar after day 15 of infection. Accordingly, survival rates during infection were not significantly different between mice injected IP or SC, with 30% of the mice dying by day 15 p.i., 40% between days 20 and 22 and the remaining dying by day 28 post-infection (Figure 3C).

**Figure 4:**
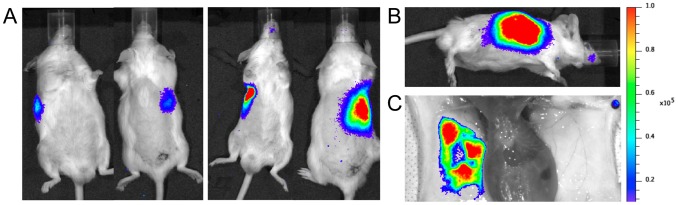
Proliferation of *T. vivax* in the skin during the prepatent period after sub-cutaneous injection. **A**. Ventral and dorsal view of a representative Outbred mouse out of two experimental groups of 12 and 14 mice injected subcutaneously with 10^2^ BSF expressing luciferase (see Figure 3). Bioluminescence was measured at days 9 (left panel) and 10 (right panel). **B**. Lateral view of the mouse on day 10. **C**. The mouse was sacrificed on day 10 and bioluminescence measured in the subcutaneous compartment. Color scale to the right of the pictures encodes for signal intensity (photons/second).

Interestingly, while detectable light emission in IP-infected mice invariably correlated with parasite appearance in peripheral blood, we noted that light emission was already detectable in SC-infected mice in the prepatent period. In efforts to investigate this phenomenon, we infected a group of mice with 10^2^ *Tv*LrDNA-luc by the SC route and monitored bioluminescence every day during the prepatent period. Light emissions were seen to increase between days 8 and 9 post-infection, suggesting that the parasite load was increasing but remained below the limit of detection of the microscopy visualization technique (<10^4^/mL). By the time parasitemia had become detectable (day 9–10), the mice showed a bright spot on the right lateral flank close to the injection site (Figure 4A) that gradually increased thereafter (Figure 4B). The mice were sacrificed on day 10 for gross anatomy and a representative mouse is shown in Figure 4C. As can be seen, the light emission is confined to the skin near the SC inoculation site. The increase in light emissions in this area and the very circumscribed foci of photons shows that the infection initially develops *in situ* and that parasite multiplication takes place in the skin close to the injection site before parasites reach the bloodstream.

### *In vivo* imaging to monitor *T. vivax* infection, quantitate parasites and map their distribution

**Figure 5:**
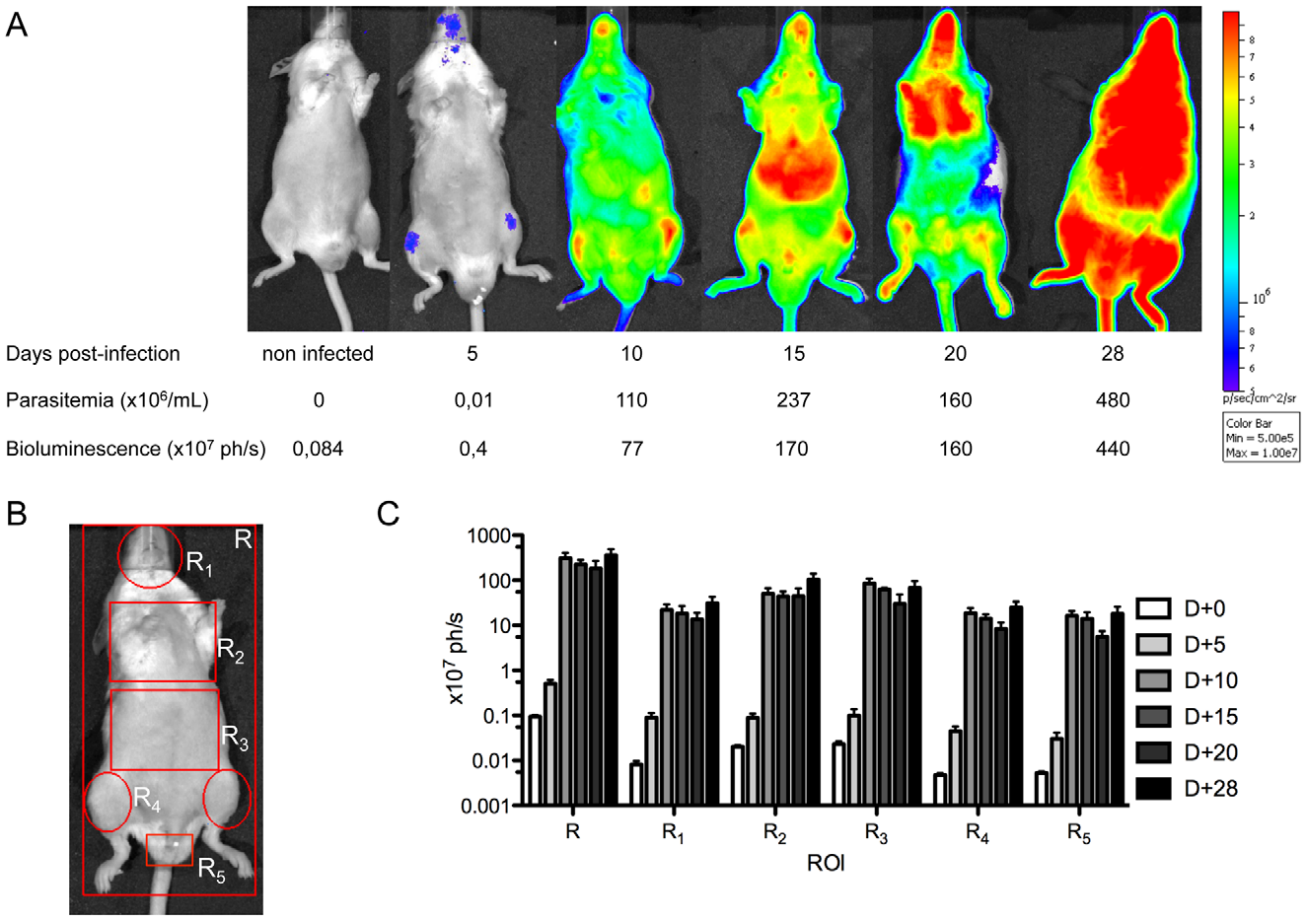
Parasite distribution in the course of the infectious process. Two groups of 16 and 18 male Outbred mice were injected IP with 10^2^ BSF. Measurements of parasite dissemination were made for 3–6 mice on different days after inoculation. **A**. Selection of a representative mice for each time point. Parasitemias (×10^6^/mL of blood) and total bioluminescence (×10^7^ photons/second) are indicated below the pictures. Color scale to the right of the pictures encodes for signal intensity (photons/second). **B**. Mouse body divided into regions (R) of interest (ROI) symbolized by red lines. **C**. Light detected throughout the infectious process (gray scale for days 0 to 28 at the right) in the different ROI examined (R, R_1_, R_2_, R_3_, R_4_, R_5_); bars express arithmetic means from 3–6 mice analyzed individually for each time point +/− SD of the means.

*T. vivax* infection was followed *in vivo* by inoculating new groups of mice by the IP route with 10^2^ *Tv*LrDNA-luc BSF and following the infection by biphotonic analysis. Mouse parasitemias were measured individually both microscopically and by light emission, and as described here above, the two techniques gave comparable results. Groups of at least 3 mice were analyzed at each time point and bioluminescence recorded individually. Light became detectable 5 days after infection and at this point was 4 fold background levels in non infected control mice. These observations correlated with very low parasitemias (1–2×10^4^ parasites/mL), as determined microscopically. Figure 5A shows the results obtained with one infected mouse representative of a group of 3 mice examined by time of infection as compared to a uninfected control during the study period of 4 weeks. The first foci observed were located at the muzzle and the inguinal regions. Once parasitemia increased (10^5^–10^6^ parasites/mL), photons were detected along the entire body, with hotspots corresponding to spleen, lungs and liver. Attempts were made to accurately define the dynamics of parasite dissemination by segmenting the images obtained for each animal into several areas corresponding to whole body (R), head (R_1_), thoracic region (R_2_), abdomen (R_3_), inguinal (R_4_) and testis areas (R5) (Figure 5B). The bioluminescence detected from day 10 for each of these defined areas was up to 10000 fold background levels (Figure 5C) for some areas. No apparent parasite infiltration/retention was seen in any particular region, supporting the notion that the development of *T. vivax* is confined to the vascular compartment.

### *T. vivax* induces systemic organ commitment and severe signs of cerebral nervous system involvement

**Figure 6:**
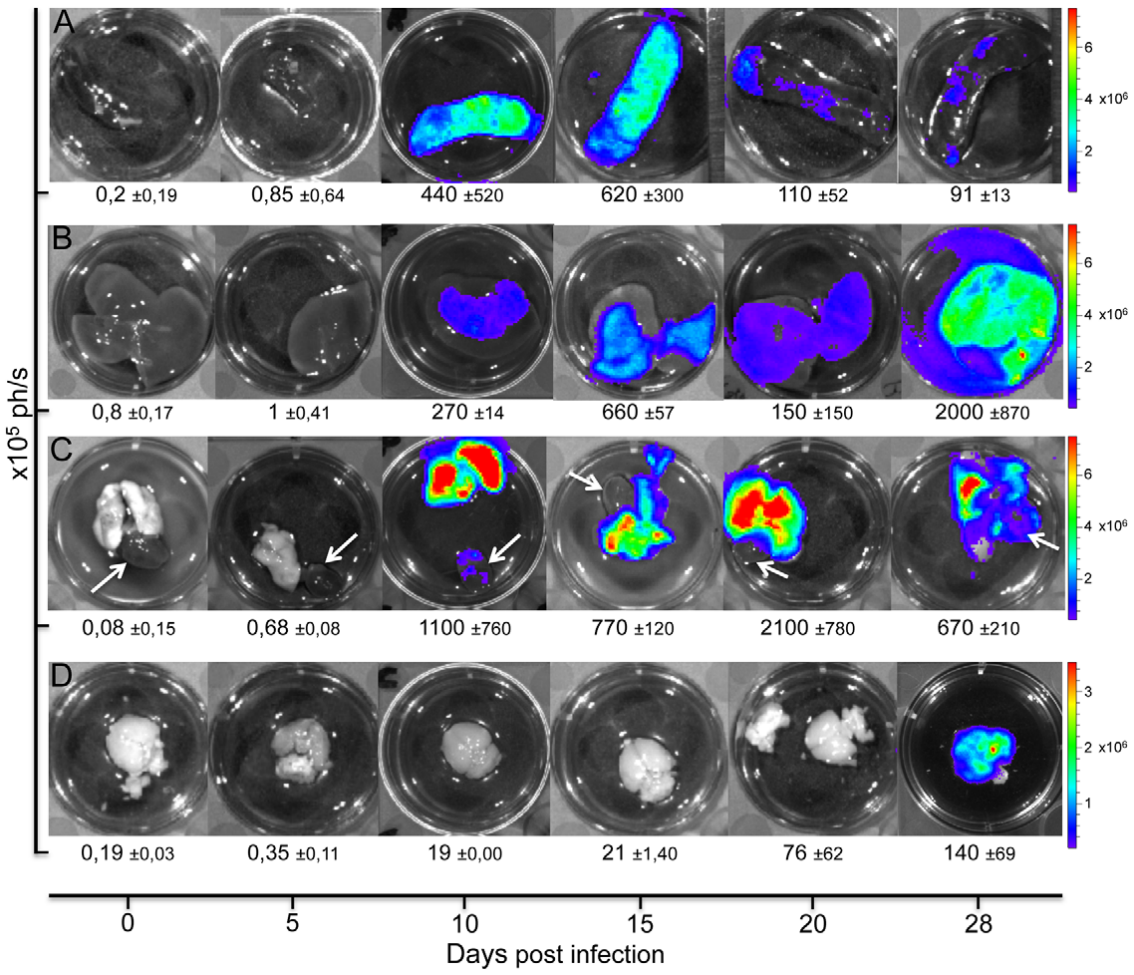
*Ex vivo* organs of infected mice confirming the systemic dissemination of the parasite and the brain commitment in late phases of infection. Detection of bioluminescence *ex-vivo* in organs isolated from mice injected intraperitoneally with 10^2^ *Tv*LrDNA-luc BSF. Organs were rinsed in PBS and D-Luciferin was added before light detection. Organs from a representative mouse out of 3 for each time point are depicted. Color scale to the right of the pictures encodes for signal intensity (photons/second). The values of each animal have been taken into consideration to obtain the arithmetic means of bioluminescence ×10^5^ ph./s ± the Standard Deviations of the means and are indicated underneath the pictures. (**A**) spleen, (**B**) liver, (**C**) lungs and heart (white arrows) and (**D**) brain. Even if individual variations between the organs pertaining to the same group of mice are observed for each time point, they do not influence the data interpretation.

In a further set of experiments we compared the distribution of *T. vivax* at key time points in the infectious process by *ex vivo* examination of the main organs affected after injection of the *Tv*LrDNA-luc strain. Groups of 3 mice per time point were sacrificed and the light emitted by spleen, lungs, liver and brain was promptly recorded in the presence of excess of D-Luciferin. As can be seen in Figure 6A and regardless the individual variation in the level of bioluminescence observed inside the group, the spleen was affected soonest after infection and constituted one of the first sites of parasitic retention (day 10 p.i., peaking by day 15 p.i.), as shown by at least 1000 fold greater light emission than in uninfected mice. Photon emission increases were recorded for all the organs tested after day 10, with elevated levels in liver and in particular lungs (Figures 6B and 6C). At about day 20 p.i., the luminescence in the lungs of infected mice accounted for up to 15% of the total signal recorded for the entire body, compared with 1% in lungs harvested from non-infected mice. Likewise, while no surprising light emission is apparent in the hearts after 10 days of infection (Figure 6C arrows), a significant rise in photons per second (1,4×10^7^±6×10^6^ ph./s, not shown) is recorded for the mouse hearts to attain more than 1000 times the background levels for the organ by day 28 of infection.

**Figure 7:**
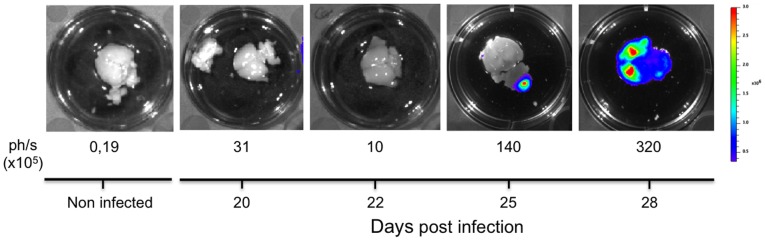
Bioluminescent *T. vivax* validates the presence of the parasite in the central nervous system in late infection phases. Detection of bioluminescence *ex-vivo* in brains isolated from mice injected intraperitoneally with 10^2^ *Tv*LrDNA-luc BSF analyzed at days 20, 22, 25 and 28 of infection as compared with a brain from a non infected mouse. Brains were rinsed in PBS and D-Luciferin was added before light detection. One brain from a representative mouse out of 3 for each time point is depicted. Color scale to the right of the picture encodes for signal intensity (photons/second). The values for each brain are indicated underneath the pictures (×10^5^ ph./s).

Use of bioluminescent *T. vivax in vivo* also allowed the validation of previous data which showed that the parasite may cross the blood brain vessels and lodge into the brain parenchyma [6], [14]. Indeed the bioluminescence signal from the brains of the animals that resisted longer the hyperparasitemia peak (20–30% survival by day 20, see Figure 3C), increases substantially from day 20 (Figure 7) but it is only visible after 25 days p.i. in localized light emission foci, just some days preceding death.

## Discussion

*T. vivax* is one of the leading parasites responsible for AAT, or *Nagana*, that still ranks among the most neglected diseases. One of its main particularities that can explain its capacity to emerge in areas free from *tsetse* flies is its ability to also be transmitted mechanically by a broad spectra of haematophagous insects [5], [15]–[17]. We have previously developed experimental models of *T. vivax* infection using mice infected with the ILRAD1392 reference strain [9]. Immunobiological and immunophysiopathological analyses confirmed that these models are reliable and consistent with all the relevant characteristics of the animal disease [9], [10]. Furthermore, we have also developed robust methods for parasite growth and differentiation in axenic cultures, paving the way to appropriate conditions for the first genetic manipulation of *T. vivax* [11]. In the study reported herein we generated an ILRAD1392 strain that stably expresses firefly luciferase (*Tv*LrDNA-luc). Then, in detailed studies using this bioluminescent parasite, we ascertained that it has the same virulence in immunocompetent mouse models and behaves in the same manner as WT parasites. We established that these *Tv*LrDNA-luc mutant parasites can be used successfully to i) monitor *in vivo* the infectious processes triggered by *T. vivax* and ii) evaluate organ infiltration by these parasites *in vivo* and *ex-vivo*. To the best of our knowledge, the study reported herein is the first to use a systematic imaging method to study the experimental model of *T. vivax* infection *in vivo* and the first to demonstrate the presence of the parasite in the brain by simple bioluminescent signal.

The use of bioluminescent parasites is a strategy of choice for investigating and following infectious processes *in vivo* [18], [19]. The approach has been used successfully to analyze infections caused by *Plasmodium berghei*, *Leishmania major*, *Toxoplasma gondii*, *Trypanosoma cruzi* and *T. brucei* [13], [20]–[28]. Here, an imaging system is used to quantify the light emitted by transfected cells - and in particular by microorganisms constitutively expressing the luciferase reporter gene - and thus monitor the infectious process *in vivo* without animal sacrifice. The technique is widely used for instance to screen new active compounds with the intention of discovering novel chemotherapies [20], [22], [29]–[31]. We decided to use the *Tv*LrDNA-luc strain to follow the dynamics of *T. vivax* infection *in vivo* from the start to the end of the infectious process. We compared the results obtained with two different routes of infection, i.e. the conventional experimental intraperitoneal (IP) route and subcutaneous inoculation that mimics natural transmission of the parasite by the vector. The only difference observed between these routes was that the subcutaneous injection resulted in a more prolonged prepatent period and in parasite multiplication *in situ* for some days before it reached the bloodstream. These observations are fully consistent with data previously obtained mainly with *T. congolense* and *T. evansi* where it was shown that the inoculation of metacyclics into the animal's hypodermis either by insect bite or syringe resulted in the development of a local inflammatory reaction, called inoculation chancre [32]–[35]. This was followed by parasite multiplication in the skin, as shown by conventional histopathology, and their migration into the bloodstream. In our study, we did not observe any inflammatory reaction at the injection site. This discrepancy could be due to the nature of the parasitic form injected since it has been previously reported that *T. congolense* bloodstream forms, at least in low numbers, are unable to induce a metacyclics-like inflammatory reaction [36].

Although it is generally recognized that African trypanosomes migrate from the skin into the blood via the lymph system, our results did not demonstrate whether or not parasite multiplication in this compartment contributes to the infection spreading or to the orientation of the immune response. However, the light emissions we measured clearly demonstrated that growing parasites were accumulating close to the inoculation site. The results we obtained with bioluminescent *T. vivax* are fully consistent with our previous reports [10] and confirm that the parasite spreads across the spleen and liver compartments. It is interesting to note that lung infiltration by the parasite is difficult or impossible to observe by immunohistopathology, contrasting with our present observations. But, considering that the bioluminescent reaction is dependent on the O_2_ concentration [37], we cannot exclude that the substantial signal in the lungs during the late phases of the infection partially results from the abundance of O_2_ in this organ. Our previous report [9] has revealed the presence of innumerous parasites in the ventricular cavities of the heart. The present data showed a considerable and unfailing increase of luminescence throughout the period of study which attains its maximum between days 15 and 20 of infection when 40% of the mice die. These observations are suggestive of a congestive heart failure-inducing death, consistent with that reported for infected cattle [38], [39]. Noteworthy, the progressive increase of bioluminescence observed in testis (R5) is suggestive that parasites can pass through Sertoli cell barrier during infection and thus contribute to reproductive disorders in the seminiferous tubules, as already suggested in reports with host infected with *T. vivax* and *T.b. brucei* [27], [40]–[42]

Our observations together with earlier histopathological studies [9] nevertheless suggest that the parasite reaches the brain tissues during late (encephalic ?) phases of the infection for those mice that survive longer the hyperparasitemia occurrence. The light emitted by the brain increased slightly up till day 20 post-infection (<400 fold the background) then rose substantially by day 25, reaching sufficient levels (up to 2000 fold the background) to provide a picture of parenchymal infiltration. These observations corroborate previous reports showing molecular and histopathological data on the detection of *T. vivax* both in the cerebrospinal fluid and the nervous tissue parenchyma of goats [14]. Correspondingly, at late stages of *T.b. brucei* infection in rat and mouse models, the parasite actively migrate out of the cerebral blood vessels, cross the endothelial basement membrane, the perivascular space and the parenchymal membrane to invade the brain parenchyma, with no signs of plasma protein leakage into the brain [43]. These results were indicative that parasites had penetrated the parenchyma through the blood brain barrier rather than from circumventricular organs or through the cerebrospinal fluid (for a review, see [44]). Our present data could not clarify if the bioluminescent signal results from intra or extra vascular parasites. Using PCR of CSF extracts and histopathology of the brain may give a better picture of this question (ongoing experiments).

Altogether, the results reported herein strongly support use of the *Tv*LrDNA-luc strain for detailed *in vivo* studies of the infectious process triggered by *T. vivax*. In particular, our data showed that the *Tv*LrDNA-luc strain is highly appropriate to ascertain the evolution of the infection and the mechanisms involved in the progression of the disease. A more in-depth comprehension of the strategies set in place by the parasite to persist inside the host could open up perspectives for the development of a new therapeutic strategy against AAT. Our data also validate the use of bioluminescent *T. vivax* in high throughput drug screening strategies.
